# Retrospective clinical study on outcome in cats with nasal planum squamous cell carcinoma treated with an accelerated radiation protocol

**DOI:** 10.1186/s12917-017-1018-3

**Published:** 2017-04-04

**Authors:** Evgeniya Gasymova, Valeria Meier, Franco Guscetti, Simona Cancedda, Malgorzata Roos, Carla Rohrer Bley

**Affiliations:** 1 Division of Radiation Oncology, Vetsuisse-Faculty, University of Zurich, CH-8057 Zurich, Switzerland; 2 Institute of Veterinary Pathology, Vetsuisse Faculty, University of Zurich, CH-8057 Zurich, Switzerland; 3 Centro Oncologico Veterinario, 40037 Sasso Marconi (BO), Italy; 4 Department of Biostatistics, Epidemiology Biostatistics and Prevention Institute, Faculty of Medicine, University of Zurich, CH-8001 Zurich, Switzerland

**Keywords:** Nasal planum, Squamous cell carcinoma, Radiation therapy, Accelerated protocol, Cat

## Abstract

**Background:**

Cutaneous squamous cell carcinoma of the nasal planum in cats is a common indication for antitumor treatment such as external beam radiation therapy. Curative-intent radiation therapy has been described as a valuable treatment option, resulting in long and stable tumor control in these patients. The aim of the current study was to evaluate outcome and toxicity, as well as possible prognostic factors using an accelerated hypofractionated radiation therapy protocol.

Cats with squamous cell carcinoma of the nasal planum treated with an accelerated radiation protocol (10 × 4.8 Gy, over one week) were retrospectively evaluated. Tumor- and treatment-associated variables were evaluated in respect to local control and survival.

**Results:**

Forty-four cats met the inclusion criteria for this study. All cats showed complete response to therapy. Median disease-free interval (DFI) for all cases was 916 days (95% CI: 456-1377). One- and two-year DFIs were 71% (95% CI: 56-86%) and 60% (95% CI: 43-77%). Of the tested variables, only tumor volume showed a tendency to influence DFI, with larger tumors having a 5.4 times greater risk of recurrence than the smaller ones (HR 1.33 (95% CI: 0.99-1.79), *p* = 0.054). Median overall survival (OS) was 902 days (95% CI: 862-942). One- and 2-year OSs were 79.3% (95% CI: 67.3-91.3) and 58.4% (95% CI: 42.8-74). Of the tested variables, again, only tumor volume influenced OS with larger tumors having a 6.3 times greater risk of dying than the smaller ones (HR 1.36 (95% CI: 1.07-1.73), *p* = 0.010). The acute and late toxicity profile was low and hence clinically acceptable.

**Conclusions:**

Curative-intent radiation therapy with an accelerated fractionation schedule can be considered a safe, cosmetically superior treatment option for cutaneous squamous cell carcinomas of the nasal planum in cats, resulting in long and stable tumor control.

## Background

Cutaneous squamous cell carcinoma (cSCC) of the nasal planum in cats is a common indication for antitumor treatment, and larger, invasive carcinomas are often treated with external beam radiation therapy. [1–4] These tumors are mostly caused by chronic exposure to sunlight (ultraviolet (UV) light) with older and particularly fair-haired cats being at higher risk. [5] As fur is a physical barrier to solar-induced lesions, the nasal planum as a non-haired and often unpigmented area represents a predilection site for cSCC. The treatment of advanced-stage lesions at this location can be challenging. [6] Precancerous (actinic) lesions can persist over months to years, and in some cats a transformation to a more malignant carcinoma in-situ and subsequent invasive variant takes place. [7] Progression is often initially slow, and the pre-malignant or superficial variants of neoplastic condition can be treated by several means, such as photodynamic therapy [8–10], strontium-90 plesiotherapy [11, 12], intralesional chemotherapy [13], curettage and diathermy. [14] However, for larger or more invasive tumors, extensive surgical removal is often a limited treatment option, due to the aesthetically disfiguring cosmetic outcome. [6, 15] Curative-intent radiation therapy has been described as a cosmetically superior option for this often rapidly destructive, painful and disfiguring variant of the disease, resulting in long and stable tumor control in these patients. [2–4].

The invasive form of the disease is thought to be a rapidly dividing tumor. [2, 3, 16] In prior publications of our group, an accelerated external beam radiation therapy protocol applied to a small group of cats with nasal planum and periocular squamous cell carcinomas was observed to lead to better responses in cases with high Ki67 reactivity, i.e. faster proliferating. [2, 3, 16] With these earlier findings in mind, this retrospective, bi-institutional case series was compiled to assess the effectiveness of the previously published 10 × 4.8 Gy accelerated radiation therapy protocol in a larger group of cats with macroscopic cSCC of the nasal planum, and to evaluate tumor and treatment variables associated with local control and survival. Our hypothesis was that the accelerated hypofractionated radiation therapy protocol would result in a clinically acceptable acute and late toxicity profile and provide a long disease free interval (DFI) and overall survival (OS).

## Methods

### Study design

Uncontrolled, single-arm retrospective clinical study.

### Patient and tumor characteristics

Medical records of cats with squamous cell carcinoma of the nasal planum admitted to the Division of Radiation Oncology of the Vetsuisse-Faculty, University of Zurich, and the Centro Oncologico Veterinario, Sasso Marconi, Bologna, between 2003 and 2015 were reviewed.

Cats were included in the study if they had a histopathologically or cytologically confirmed diagnosis of cSCC of the nasal planum and underwent 10 × 4.8 Gy radiation therapy protocol in a macroscopic setting. The outcomes of patients with cSCC of the nasal planum reported in a previous study by our group (*n* = 12) were reassessed over a longer follow-up period and included as well. [3] Adequate available follow-up consisting of tumor response, date of recurrence or systemic progression as well as date and cause of death was required.

Clinical staging for regional and distant disease consisted of physical examination, routine complete blood count and serum biochemistry, fine-needle aspiration of enlarged regional lymph nodes and thoracic radiographs. Stage was divided into groups with a modified TNM staging system: T1 was comprised of lesions measuring <1.5 cm in diameter, T2 consisted of neoplasms >1.5 cm in diameter; substage a: non-invasive, substage b: invasive (each based on histopathology or visual assessment). [17] Medical records were carefully studied in order to retrieve further information such as: signalment (age, sex, breed), concurrent diseases, presentation (primary versus recurrent), number of previous treatments, involved site, tumor size, histology, clinical stage, response to radiation therapy, time to disease progression, treatment-related side effects or complications, time and cause of death, and date of last follow-up visit. In addition, Ki67 levels were evaluated for prognostic influence. Pathologic diagnoses and grading were made by an experienced pathologist (FG) as described previously. [3, 18] In brief, three grades (I, II, and III) were distinguished, with more malignant morphology indicated by higher grades. The following characteristics were scored based on a scale: lack of differentiation and of keratinization, mitotic rate, and nuclear polymorphism. A proliferation score was determined by means of immunohistochemistry for Ki67 (MIB-1 antigen) as previously described. [3] In brief, digital images from several randomly chosen regions of each tumor were reviewed and 600–800 cells were counted, each. The percentage of positive cells was determined and resulted in the following scores: Score 0 was defined as 0–4% positive cells, score 1 as 5–19%, score 2 as 20–59%, and score 3 as more than 60% positive cells. Tumor size before radiation therapy was evaluated by caliper measurement during physical examination. Tumor volume was calculated by the rotational ellipse method (length x width x depth x π/6).

### Treatment

All cats were treated with external beam megavoltage radiation therapy. Radiation was delivered with a 6 MV (MV) linear accelerator (Dynaray LA20; ABB, Clinac DMX or Clinac iX, Varian, Palo Alto, USA) using electrons. Positive lymph nodes were either surgically removed later (*n* = 1) or irradiated in a second field (*n* = 1). Treatment planning was performed by hand calculation, performed or checked by the same radiation oncologist at both institutions (CRB). For the treatments, some of the cats were positioned into a custom-made bite block for reproducible immobilization. [19] The GTV (gross tumor volume) was defined as the macroscopically visible part of the lesion and a CTV (clinical target volume), accounting for subclinical microscopic disease extension of 5 mm (presumed local infiltration). The CTV-margin was then extended three-dimensionally by approximately 1 cm to define the planning target volume (PTV), accounting for patient motion, and setup uncertainties. The closest available electron insert (4 × 4 or 6 × 6 cm) was used (diagonally, e.g. the long axis of the field in line with the cranial-caudal axis of the patient).

The recommendations for specifying dose and volumes were adhered to as proposed in the relevant veterinary medical literature. [20, 21] The according fields (field size of 4×4cm or 6×6cm) were applied at 100 cm source surface distance (SSD) and energies were chosen to adequately cover the PTV. Bolus was used to ensure dose homogeneity and sufficient dose-build up at the surface. The 90% isodose line was chosen to encompass this target volume and for dose normalization. [22] The prescribed dose was 48 Gy delivered in 10 fractions of 4.8 Gy applied twice per day with an interval of 6 h or more (allowing for normal tissue repair), resulting in an overall treatment time of 5 consecutive days.

### Toxicity

The Veterinary Radiation Therapy Oncology Group (VRTOG) scheme was used for radiation related toxicity assessment at each treatment and 2-3 weeks after treatment. As further follow-up, monthly rechecks were recommended. [23] Specific attention was paid to wound-healing complications in the acute setting, as well as vascular or osseous complications and second malignancies in the late setting.

### Outcome and follow-up

Outcome information was obtained by the study of medical records or regular phone communication with referring veterinarians and owners at the end of the study period. Follow-up care included a medical history and physical examination at progressive intervals beyond treatment. Response data was noted in a modified version in adherence to response evaluation criteria in solid tumors (RECIST) guidelines for dogs. [24] Complete remission (CR) was defined as the disappearance of the target lesion. Partial response (PR) was defined as a reduction of at least 30% in the sum of diameters of target lesions from baseline. Stable disease (SD) was defined as <30% decrease or <20% increase in the sum of diameters of target lesions from the smallest sum while on treatment. Progressive disease (PD) was defined as an increase in the sum of diameters of target lesions by at least 20% over the size present at entry on study, the appearance of new lesions, such as metastatic regional lymph nodes or distant, pulmonary metastases. Responses were required to last for at least one month. Follow-up imaging was based on individual risk or concurrent problems, eventual symptoms, and/or clinicians’ and/or owners’ preferences, and included thoracic radiographs and abdominal ultrasound.

### Statistical analysis

Statistical evaluation was performed under the supervision of a biostatistician (M.R.) and computed with a commercial statistical software package (IBM® SPSS® Statistics, Version 23). Description of quantitative data characteristics, other than disease-free interval (DFI) and overall survival (OS), is given by mean (± SD), unless otherwise specified. Description of qualitative characteristics is provided in absolute and relative frequencies.

DFI was defined as the interval between the last day of radiation therapy to measurable progression of disease. OS was defined as the interval between the first fraction of radiation therapy and death. Cats that were still free of progression at the time of data evaluation were censored for DFI analyses. For OS, all deaths were considered events and cats that were still alive at the time of data evaluation or lost to follow-up were censored. Kaplan-Meier survival analysis was used and followed by Logrank or Breslow-Gehan-Wilcoxon tests. In the absence of crossing of survival curves, the Logrank test was applied. Otherwise the Breslow-Gehan-Wilcoxon test was used. The univariate and multiple Cox-regression analysis was used to determine whether the following factors were significantly associated with DFI or OS: age, weight, sex, histological grade (I versus II versus III), tumor size, tumor stage (and substage), Ki67 proliferation index, as well as the pre-treatment hematologic parameters (hemoglobin (Hb) and packed cell volume (PCV)). Distribution in tumor volumes were skewed; thus logarithmically transformed values were used rather than raw measurements (lnVol). Survival estimates are presented as median with the corresponding 95% confidence intervals (95% CI). Moreover, Hazard-Ratio (HR) together with the corresponding 95% CI is reported. Results of statistical analyses with *p*-value less than 0.05 were interpreted as statistically significant.

## Results

### Patient and tumor characteristics

Forty-four cats met the inclusion criteria for this study. Twenty-one of the cats were female (19 spayed) and 23 were male (22 neutered). The cats were mostly domestic short-hair (*n* = 41), 2 were of Angora breed and 1 Turkish van. The ages ranged from 5 to 16 years with a mean of 11.3 (± 2.7) years. Body weight ranged from 2.9–7.4 kg, with a mean of 4.7 (± 1.1) kg. Thirty-four animals were treated at the Division of Radiation Oncology of the Vetsuisse-Faculty, University of Zurich, Switzerland and 10 animals at the Centro Oncologico Veterinario, Sasso Marconi, Bologna, Italy.

One cat had two prior surgical tumor removals. Packed cell volume ranged from 19 to 49.7% with a mean of 34.0% (± 7.3), (*n* = 44). Hemoglobin ranged from 5.9-15.2 g/dl, with a mean of 11.0 g/dl (± 2.4), (*n* = 43). Pretreatment tumor volumes were 0.2 to 70.6 mm^3^, with a mean of 15.0 mm^3^ (± 19.1), and transformed volumes (lnVol) -1.7 to 4.3, with a mean of 1.6 (± 1.7). Regarding stage, 18.2% (*n* = 8) were stage T1a, 38.6% (*n* = 17) T1b, 4.5% (*n* = 2) T2a and 38.6% (*n* = 17) T2b tumors. Diagnosis of cSCC was histologically confirmed in 84% (*n* = 37) and cytologically in 16% (*n* = 7). Of the 44 cases, two had cytologically confirmed positive lymph nodes (4.5%) at the time of tumor diagnosis, none of the cats presented with distant metastasis. Of these two cats, one had lymph node excision two months after radiation therapy, in the other cat, the lymphnode was irradiated in a second treatment field. It was possible to assign histological grade in 54.5% of the cases (*n* = 24). Of these tumors, 20.8% were of grade 1 (*n* = 5), 50% of grade 2 (*n* = 12), and 29.2% of grade 3 (*n* = 7). For the remaining 20 cases, the histological samples were not retrievable; hence grade could not be assessed. Ki67 positivity could be evaluated in 23 cats and ranged from 36.7-81.6% with a mean of 53.2% (±13.6). When described as a score, 15 cats had a Ki67 score 2 and 8 cats a score 3 (Table 1).

**Table 1:** Overview of all treated cats included in the study

Nr	Age [years]	Weight [kg]	PCV [%]	Hb [g/dl]	TNM	Grade	Ki 67 [% positive cells]	Ki 67 [score]	DFI [days]	OS [days]
1^a^	11.0	3.3	36.0		T2bN1M0	3	60.6	3	1′432	1′436
2^a^	8.0	3.3	42.0	12.7	T1bN0M0	2	36.7	2	365	1′227
3^a^	11.0	4.9	24.0	8.6	T2bN0M0	2	58.7	2	916	1′017
4^a^	12.0	5.7	34.0	8.6	T1aN0M0	2	50.2	2	456^b^	460
5^a^	12.0	3.0	27.0	9.1	T2bN0M0	2	45.3	2	913^b^	917^b^
6^a^	8.5	6.3	35.0	10.2	T1bN0M0	2	63.8	3	2′173^b^	2′177
7^a^	8.0	4.7	42.0	14.2	T1aN0M0	2	42.7	2	87	124
8^a^	14.0	3.1	21.0	7.0	T2bN0M0	1	46.3	2	139	198
9^a^	8.7	5.1	24.0	8.2	T1aN0M0	1	74.9	3	2′155	2′423
10^a^	9.5	4.0	29.0	11.6	T2aN0M0	3	81.6	3	656	900
11	12.0	6.9	27.0	8.9	T1bN0M0	2	43.9	2	530^b^	534
12^a^	15.0	4.6	27.0	8.6	T1bN0M0	3	71	3	1′721^b^	1′725
13^a^		4.0	34.0	11.6	T2bN0M0	2	52.7	2	266^b^	270
14	11.0	4.3	23.0	8.3	T2bN0M0	1	69.5	3	67	74
15	13.0	5.1	39.0	11.8	T1aN0M0	2	51.8	2	538^b^	542^b^
16	14.0	3.7	36.0	12.7	T1aN0M0				177	874
17	15.0	7.4	33.0	10.9	T1bN0M0	3	41.4	2	858	902
18	11.0	3.6	44.0	15.2	T2bN0M0	3	61.4	3	271	277
19	9.0	4.9	33.0	10.5	T2bN0M0	1	40.4	2	208	362
20	12.0	4.0	31.0	11.0	T2bN0M0				363	367
21	7.0	4.1	44.0	15.0	T2bN0M0				351	1′514
22	15.0	3.8	30.0	10.5	T1bN0M0	2	71.8	3	726^b^	730^b^
23	11.0	4.2	26.0	8.7	T1bN0M0	3	38.1	2	956^b^	960^b^
24	11.0	5.6	32.6	6.0	T2bN1M0	2	42.2	2	358	472
25	15.0	4.3	19.0	5.9	T2bNxM0	3	38.5	2	339	490
26	14.0	5.0	36.0	12.4	T2bN0M0				641	659
27	10.0	3.9	30.0	10.0	T1bNxM0	1			482^b^	486^b^
28	11.0	3.9	32.0	11.1	T1bN0M0				473	570
29	14.0	3.4	26.0	8.7	T2bN0M0				1′460^b^	1′464
30	13.0	4.9	39.0	13.3	T2bN0M0				226	230
31	8.0	4.0	36.0	12.8	T2bN0M0				466^b^	470
32	13.0	5.0	44.2	14.8	T1aN0M0				356^b^	360^b^
33	11.2	6.1	43.0	14.1	T1aN0M0				808^b^	812^b^
34	16.0	2.9	45.3	13.6	T2bN0M0				346^b^	350^b^
35	14.0	7.3	45.1	14.1	T2bN0M0				184^b^	189
36	14.0	4.2	29.3	9.6	T2bN0M0				17^b^	22
37	11.0	3.7	49.7	14.9	T1aN0M0				1′078	1′082
38	9.0	5.6	34.6	10.1	T2bN0M0				153	195
39	9.0	6.2	37.0	13.0	T2bN0M0				1′890^b^	1′894^b^
40	14.0	4.7	41.9	13.1	T1bN0M0				452^b^	452^b^
41	8.0	4.6	36.0	10.9	T1bN0M0	2	40.9	2	728^b^	732^b^
42	6.0	5.5	30.7	10.8	T2bN0M0				347^b^	351^b^
43	5.0	5.0	32.5	10.3	T1bN0M0				902^b^	906
44	12.0	6.1	37.0	9.9	T2aN0M0				487^b^	492

*PCV* Packed cell volume, *Hb* Hemoglobin, *TNM* Modified staging system, *x* Missing data, *DFI* Disease free interval, *OS* Overall survival, ^a^cats previously reported [3], with a longer follow-up; ^b^All deaths were considered events; cats still free of progression, alive at the time of data evaluation or lost to follow-up were censored

### Treatment protocol, side effects

All cats were treated with single electron fields with energies ranging from 6 to 9 MeV, 6 MeV was used in 40 cats (90.1%) and 9 MeV in 4 cats (9%). Acute side effects were assessed in 39 cases and consisted of grade 0 toxicity in 3 cats (7.7%), grade 1 toxicity (erythema, dry desquamation, alopecia or epilation) in 23 cats (59%), grade 2 toxicity (patchy, moist desquamation, without edema) in 11 cats (28.2%), and grade 3 toxicity (confluent moist desquamation with edema and/or ulceration) in two cats (5.1%). Late effects were assessed in 39 cases with no toxicity in 13 cats (34.2%), grade 1 toxicity (alopecia, leukotrichia) in 22 cats (57.9%), and grade 3 toxicity (necrosis, panophthalmitis, blindness) in 3 cats (7.9%). Each of these 3 cats showed different late effects, which manifested as maxillary bone necrosis, chronic moderate stomatitis and glaucoma secondary to panophthalmitis (uveitis) (the latter developed in a patient with a small tumor, without prior acute reactions in this eye and with no known association to a large treatment field) in one cat, each.

### Response to treatment

**Fig. 1:**
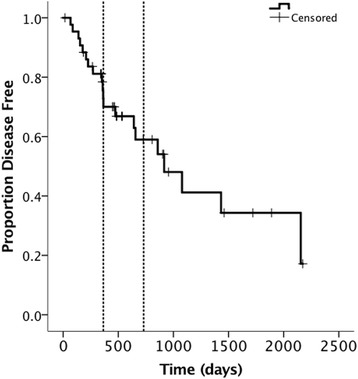
Disease free interval. The vertical dashed lines indicate 1 and 2 years

Median follow-up time for censored cases (*n* = 12, still alive or lost to follow-up) was 659 days (range 350–1894 days). Of the 12 censored cases 7 cats were still alive at the time of analysis (range 350–1894 days), and 5 were lost to follow-up (range 360-917 days). Of the 32 animals that were known to have died, 19 (65.6%) died of tumor-related or unknown (*n* = 2) reasons and 11 (34.4%) died of tumor-unrelated causes. At the first evaluation at 2-3 weeks after finishing radiation therapy, response evaluation was performed in all cats. All tumors (100%) had a complete local response (with one cat having the untreated lymphnode removed after 8 weeks of RT). Median disease free interval for all cases was 916 days (95% CI: 456–1377) (30.1 months). One- and 2-year DFIs were 71% (95% CI: 56–86) and 60% (95% CI: 43–77), Fig. 1. No difference was seen in DFI between the patients treated at the different institutions (*p* = 0.249). Of the tested variables, only tumor volume (lnVol) showed the highest tendency to influence DFI, with larger tumors having a 5.4-times greater risk of recurrence than the smaller ones (HR 1.33 (95% CI: 0.99–1.79), *p* = 0.054).

**Fig. 2:**
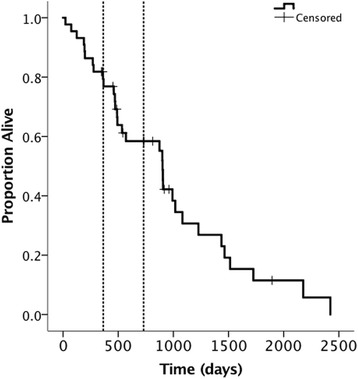
Overall survival. The vertical dashed lines indicate 1 and 2 years

Median overall survival was 902 days (95% CI: 862–942) (29.6 months). One- and 2-year OSs were 79.3% (95% CI: 67.3–91.3) and 58.4% (95% CI: 42.8–74), Fig. 2. Between the patients treated at the different institutions, no difference was seen in OS (*p* = 0.293). Of the tested variables, again, only tumor volume (lnVol) influenced OS with larger tumors having an 6.3-times greater risk of dying than the smaller ones (HR 1.36 (95% CI: 1.07–1.73), *p* = 0.010). Eight of the patients (18.1%) received an additional treatment at the time of tumor progression, consisting of photodynamic therapy (*n* = 5) or prednisone (*n* = 3). This additional treatment prolonged survival for a mean of 137 days (range 15-264; 95% CI: 64–210 days).

On multiple Cox-regression analysis, no further associations other than of tumor volume were found to influence DFI or OS.

## Discussion

Prior work has documented the effectiveness of ionizing radiation for treatment of feline nasal cSCC, resulting in partial or complete response in 93–100% and reported median progression free times of 16.5 (±3.6) months, (mean 27.6 (±3.4)) [4] with 1-year disease free survivals of 60.1% (±5.5%) [4], and estimated 1-year disease free survivals of 64% [2] and about 58% [3]. Similar to the present study, these numbers were determined in a retrospective manner, with various differences regarding radiotherapy techniques and protocols; hence a comparative interpretation of outcomes must be undertaken with care. The accelerated protocol reported here resulted in a fast and complete resolution of disease in all described cats. While the patients experiencing complete remission are not reported in one study [4], proton irradiation led to a complete remission in only 60% of the cases. [2] While the median DFI of 30.1 months (95% CI: 15–45.2 months) in this study is longer than previously reported [4], the 1- and 2-year DFIs of 71% (95% CI: 56–86) and 60% (95% CI: 43–77) are comparable with other studies, as their 1- and 2-year DFIs lie within our 95%CI range. Also, the overall survival with a median of 29.6 months in this study (95% CI: 28.3–30.9 months) and the 1- and 2-year survival of 79.3% (95% CI: 67.3–91.3 and 58.4% (95% CI: 42.8–74), respectively, are comparable to reported findings [2]. Overall survival has to be interpreted with care, as it is not an ideal readout of treatment efficacy in this disease. The patients of our cohort that received additional treatments upon recurrence lived a median 4.5 months longer.

Comparable to as described in other publications, radiation treatment was well tolerated by most of the patients in this study, with clinically acceptable acute and few long-term effects. [2–4] However, due to the retrospective nature of the study, some of the mild late toxicities such as alopecia could have been underreported. Only two cats had acute toxicity of grade 3 and they were of short duration. Three cats (7.7%) had suspected grade 3 late toxicity, which manifested in maxillary bone necrosis, chronic moderate stomatitis and glaucoma, in one case each. These side effects were rated to their highest degree and readily attributed to radiation therapy, despite the possibility of other underlying causes, such as for instance chronic infections due to dental disease or viral causes. However, it is expected that the accelerated delivery of dose leads to more severe acute side effects and the high dose per fraction and the higher incidence of acute effects can increase the risk of (consequential) late toxicity. [25, 26] It has to be taken into consideration that the onset of late toxicity has a time dependency and that the development of late adverse effects observed in our study will correspond better to the true incidence (compared to earlier studies) due to the longer follow-up period in this group of cats.

Compared to the outcome of the previous work of our group [2] the DFI in this study was significantly longer due to longer follow up time, as most of the original cats could be followed until death.

Data from the present study did not support previous observations indicating that higher proliferation rates (determined either with Ki67 or with PCNA immunohistochemistry) might be associated with longer DFIs. [3, 4] Since these two prior studies used two different methods (Ki67 vs. PCNA) an exact comparison cannot be made. Possibly due to the small number of cases the proliferative fraction assessed with Ki67 in our study had no prognostic significance. Or the prognostic significance of Ki67 truly does not exist. In contrast, tumor volume was found to be associated with shorter relapse-free periods and remains the only prognostic factor in our findings. [4] However Ki67 proliferation rate in this study was high for most of the cases and ranged from 36.7-81.6%, which suggests the aggressive nature of the tumors and can still justify the accelerated treatment approach.

Due to the use of archival data and considering the difficulty of exact clinical assessment of late toxicities, the authors may not have been able to describe the full range of responses in these cats. In addition, information gathered by telephone follow-up could have possibly skewed the described results by owner’s perception. DFIs might have been overstated as many of the animals were seen on progression and the exact onset of disease progression might have been observed incorrectly by the owner. The bi-institutionality of the study is of a lesser concern, as the treatment planning (e.g. the electron hand calculations) was done under the direct supervision of one radiation oncologist (CRB). Further limitations were that the grading was not available for every patient and that some biopsy specimens available were small-sized.

## Conclusions

Curative-intent radiation therapy with an accelerated fractionation schedule can be considered a safe, cosmetically superior treatment option for invasive cSCC of the nasal planum in cats. Considering that this treatment regime results in long and stable tumor control and can be conveniently delivered in a one-week period, it can be further recommended as current standard treatment.

## Abbreviations

CR: Complete remission
cSCC: Cutaneous squamous cell carcinoma
CTV: Clinical target volume
DFI: Disease-free interval
GTV: Gross tumor volume
Gy: Gray
Hb: Hemoglobin
MeV: Mega electron volt
MV: Megavolt
OS: Overall survival
PCV: Packed cell volume
PD: Progressive disease
PR: Partial response
PTV: Planning target volume
RT: Radiation therapy
SD: Stable disease
SSD: Source surface distance
TNM: Tumor – Node – (distant) Metastasis
UV: Ultraviolet
